# The ecological and etiological investigation of ticks and rodents in China: results from an ongoing surveillance study in Zhejiang Province

**DOI:** 10.3389/fvets.2023.1268440

**Published:** 2023-11-28

**Authors:** Jinna Wang, Mingyu Luo, Tianqi Li, Ying Liu, Guoqin Jiang, Yuyan Wu, Qinmei Liu, Zhenyu Gong, Jimin Sun

**Affiliations:** ^1^Zhejiang Provincial Center for Disease Control and Prevention, Hangzhou, China; ^2^Shaoxing Center for Disease Control and Prevention, Shaoxing, China

**Keywords:** tick, rodent, density, SFTS, hantavirus, *Leptospira*, *Orientia tsutsugamushi*, *Yersinia pestis*

## Abstract

**Objectives:**

This study aimed to analyze the population density of vector ticks and reservoir hosts rodents, and to investigate the relevant pathogen infection in Zhejiang Province, China.

**Methods:**

In this surveillance study, the data of ticks density were collected with the tick picking method on animal body surface and the drag-flag method, while the rodent density with the night trapping method. The samples of ticks were examined for the severe fever with thrombocytopenia syndrome virus (SFTSV), and blood serum and organs from rodents were subjected for SFTSV, hantavirus, *Leptospira*, *Orientia tsutsugamushi* (*O. tsutsugamushi*) and *Yersinia pestis* (*Y. pestis*) screening in the laboratory.

**Results:**

From 2017 to 2022 in Zhejiang Province, 16,230 parasitic ticks were found in 1848 positive animals, with the density of parasitic ticks of 1.29 ticks per host animal, and a total of 5,201 questing ticks were captured from 1,140,910 meters of vegetation distance with the questing tick density of 0.46 ticks/flag·100 m. *Haemaphysalis longicornis* (*H. longicornis*) was the major species. A total of 2,187,739 mousetraps were distributed and 12,705 rodents were trapped, with the density of 0.58 per 100 trap-nights. *Rattus norvegicus* was the major species. For SFTSV screening, two groups nymphal ticks of *H. longicornis* were tested to be positive. For the rodents samples, the *Leptospira* had a positive rate of 12.28% (197/1604), the hantavirus was 1.00% (16/1604), and the *O. tsutsugamushi* was 0.15% (2/1332). No positive results were found with SFTSV and *Y. pestis* in the rodents samples.

**Conclusion:**

Findings from this study indicated that the ticks and rodents were widely distributed in Zhejiang Province. Particularly, the positive detection of SFTSV, *Leptospira*, hantavirus and *O. tsutsugamushi* in ticks or rodents from this area suggested that more attention should be paid to the possibilities of relevant vector-borne diseases occurrence.

## Introduction

Current evidence show that ticks are the most important vectors of severe fever with thrombocytopenia syndrome (SFTS), and the rodents might be the possible reservoir host animals (1). SFTS is an emerging infectious disease with a relatively high fatality rate of up to 30% caused by the SFTS virus (SFTSV), a phlebovirus in the family Bunyaviridae (2). Since firstly discovered in rural areas of central China in 2009, the epidemic focus has continuously expanded (3), which posed an enormous threat to the public health. Zhejiang Province was one of the five provinces with the highest SFTS incidence in China during 2011–2016, and had reported the highest SFTS incidence in the Yangtze River Delta region of southeast China (4). Except for SFTS, rodents can also play important roles in the transmission of other diseases, such as the hemorrhagic fever with renal syndrome (HFRS), leptospirosis, scrub typhus, and plague, etc. HFRS is a rodent-borne endemic disease caused by hantavirus (5), leading to fever, hemorrhage, headache, back pain, abdominal pain, acute renal dysfunction, and hypotension (6), which is widely spread throughout China (7). Specifically, Zhejiang Province was among the six provinces with the heightened incidence rate greater than 1 per 100,000 population annually (8). *Yersinia pestis* (*Y. pestis*) is the bacterial causative agent of plague, a very serious rodent-borne disease, and the lethality of septicaemic and pneumonic plague is extremely high even almost 100% following the onset of symptoms without intensive treatment (9). Plague was considered to be endemic in restricted areas for several hundred or even thousands of years, and consistently, Zhejiang Province was always the plague epidemic focus in history (10). Leptospirosis is a major zoonotic disease worldwide, caused by pathogenic spirochete bacteria of the genus *Leptospira*, which approximately affected 1.03 million people and caused 58,900 deaths annually (9). Rodents are known with a considerably high prevalence of *Leptospira*, and play crucial effect in the spread of leptospirosis (11). According to the surveillance data from 2010 to 2014, the reported leptospirosis cases were distributed broadly across the south of China (12), and specifically, which was distributed in 9 of the 11 prefecture-level city of Zhejiang Province from 2005 to 2014 (13), suggesting a relatively wide distribution. The scrub typhus was a life-threatening human disease caused by *Orientia tsutsugamushi* (*O. tsutsugamushi*), and the rodents served as the animal reservoirs (14). The scrub typhus has expanded to all the provinces across both rural and urban areas in China, with the average annual incidence increasing sharply from 0.09/100000 population in 2006 to 1.60/100,000 population in 2016 (15). A 45 years population-based surveillance study in Zhejiang Province showed that the scrub typhus incidence declined since 1959, remained low from 1967 to 1989, and then exponentially increased after 2006 (16), indicating a re-emergence of this disease.

The population density of the vector or host animals play an important role in the transmission of natural focus infection disease. A prior study found that presence of ticks in working areas or around the house was one of the four environmental factors of SFTSV infection (17). Exposure to ticks, tick bites, presence of ticks in the residential areas or workplace and presence of rodents etc. were correlated to laboratory diagnosis with SFTSV infection (18). Similarly, for the rodent-borne diseases, studies found that changes in rodent abundance potentially drove HFRS outbreak, and HFRS cases were positively correlated with rodent density with or without a 2 months lag (19, 20). In addition to the density, the species which had different pathogen positive rate also played important impacts on the distribution of the disease. For example, researches found that the Norway/brown rat and the black rat were the most important sources of *Leptospira* infection (11). Thus, the ecological investigation of the vector and host animals was necessary to understand the possible diseases distribution and conduct early warning combined with the etiological surveillance.

Zhejiang Province is located in the southeast of China, with the subtropical monsoon climate. The natural environment in this climate is suitable for the survival of vectors and host animals, resulting in the long-term prevalence of vector-related infectious diseases. As an epidemic focus of SFTS, HFRS, leptospirosis and the historical plague areas, there was extremely high arthropod-vector-borne infectious diseases burden in Zhejiang Province (4, 10, 13, 16). Thus, the investigation of the density and pathogen carrying status of ticks and rodents was warranted for the prevention and control of the vector-related infectious diseases. In this study, we analyzed the population density of vector ticks and reservoir hosts rodents and investigated the relevant pathogen infection in Zhejiang Province, to shed light on providing the early warning and relevant control measures.

## Materials and methods

### Study design

An active density surveillance study by Zhejiang Provincial Center for Disease Control and Prevention (CDC) including ticks and rodents was performed from January 2017 to December 2022 in all the 11 prefecture-level cities (Hangzhou, Huzhou, Jiaxing, Jinhua, Lishui, Ningbo, Quzhou, Shaoxing, Taizhou, Wenzhou and Zhoushan) in Zhejiang Province, China. The ticks pathogen screening was carried out in all cities from January 2022 to June 2023. Besides, the rodents pathogen screening was performed in eight prefecture-level cities (Hangzhou, Huzhou, Ningbo, Jinhua, Zhoushan, Lishui, Shaoxing, and Taizhou) in 2022, which covered the major habitat area including plains, mountains, and islands in Zhejiang Province. The ethics committee approved the procedures for verbal consent because Zhejiang CDC has the authority of the Zhejiang provincial government to collect the related information, which is part of the disease surveillance work. This surveillance mainly involved the density and etiological detection of wild mice and ticks, and documentation of consent was not required.

### Tick surveillance

Tick surveillance was carried out four times (March, May, July, September) annually. At least 10 animals including sheep, cattle, dogs, etc. in urban and rural areas were examined for the parasitic ticks each monitoring site, with all the found ticks were collected. The drag-flag method was used to monitor the questing ticks from vegetation, which was implemented by dragging the flag slowly through vegetation for at least 30 min. All the attached ticks were removed and collected from the flag per 10 m walk. The size of the flag was 90 cm × 60 cm made with woolen flannel cloth (21). Ticks collected from the same sampled sites or animals were placed in the same tube with a unique number to identify the date, location, collection site, etc. All the ticks were taken to the laboratory. The specimens for identification and classification were stored at 75% alcohol, and identification was performed under a type microscope according to the identification pictures of common medical vectors (22). The tick specimens caught in 2022 were mainly used for etiological testing, which were stored in a freezer at −70°C in the laboratory before classification and examination.

### Rodent surveillance

The rodent surveillance was conducted in all the districts and counties of the 11 prefecture-level cities in January, March, May, July, September and November each year in Zhejiang Province, China. According to the National Vector Monitoring Program of China CDC, combined with the geographical characteristics of Zhejiang Province, three types of monitoring habitats closed to human settlement and suitable for rodents survival were chosen for the surveillance, including the urban residential areas (e.g., urban villages, urban and rural fringe areas, urban community, etc.), the key industries (e.g., catering places, food production and sales sites, construction sites, slaughter houses, brewing plants, etc.) and rural residential areas (e.g., the village, the farmland, the mountainous region and shrubbery, etc.). The medium mouse traps, mouse cages or sticky mouse boards were distributed every 15 m^2^ indoors or every 5 m along the wall root in rooms over 100 m^2^. On the farmland, the mouse traps or mouse cages were placed every 5 m, with a row spacing not less than 50 m. At least 200 mousetraps were placed in each monitoring habitat, and a total of 600 mousetraps were placed in each district or county each monitoring. The surveillance should not be carried out in the same area within three months, and the distance between the monitoring areas selected in different months should be greater than 250 m. All the traps were placed at dusk and taken back in the morning. The rodents captured were taken to the laboratory, and anesthetized in a closed container with ether or chloroform for about 10 min to prevent the escape and bite of various parasites on the rodents body surface, then the rodents were identified morphologically according to the identification pictures of common medical vectors (22). The information of date, location and collection site was also recorded.

### Pathogen screening

The samples of parasitic ticks and questing ticks were collected mainly in the tick surveillance in 2022, and additional collection was also conducted especially in the SFTS epidemic area and near the patients’ home or activity location. All the collected ticks were identified morphologically and pooled according to species, location, host animals and developmental stages (engorged or non-engorged larvae, nymphs, female and male adults). The samples of rodents were partly collected by rodents monitoring in 2022, and additional collection was also conducted for the sufficient sample size. The rodent samples were necropsied in the laboratory, then the blood serum and the organs including the liver, spleen, lung, and kidney were collected. All the samples were stored in a freezer at −70°C in the laboratory before detection.

SFTSV were tested in the samples of ticks and rodents (liver, spleen and lung). Besides, hantavirus was examined in the lung, the *Leptospira* and *O. tsutsugamushi* were examined in the liver, spleen and kidney of the rodents. The samples were homogenized and centrifuged. Total nucleic acids (including RNA and DNA) were extracted from the homogenates by using a Magnetic Viral DNA/RNA Fast Kit (TIANGEN, China) according to the manufacturer’s instructions. Then real-time fluorescence quantitative PCR assay were performed for target genes. For the *Leptospira* and *O. tsutsugamushi*, the Takara PrimeScript^™^ one step RT-PCR kit (Takara, Japan) was used, and the total volume of the reaction system was 20 μL, which included Taq DNA polymerase and dNTP mixture qPCR Master Mix 10 μL, probe 0.4 μL (final concentration 200 nmol/L), upstream and downstream primers 0.8 μL (final concentration 400 nmol/L), DNA template 3–5 μL, deionized water complement. The cycling parameters included denaturation at 95°C for 5 min (one cycle), amplification at 95°C for 15 s and 60°C for 45 s (40 cycles). The total volume of the reaction system of hantavirus and SFTSV was 25 μL, including 2 × Reaction Mix 12.5 μL, probe (10 μM) 0.3 μL, upstream and downstream primers (10 μM) 0.5 μL, RNA template 5 μL, Enzyme mix 1.0 μL, and deionized water supplement. The cycling parameters included 50°C for 30 min (one cycle), 95°C for 10 min (one cycle), 95°C for 15 s and 60°C for 45 s (40 cycles). Cycle threshold values of *Leptospira*, hantavirus and SFTSV were ≤35, and *O. tsutsugamushi* was ≤33, respectively. The relevant target genes (Table 1) were provided by China CDC and according to the research of Wu et al. (23). Data were analyzed using the software supplied by the manufacturer.

**Table 1:** Primers and probes for the pathogens screening.

Pathogens	Primers and probes	Sequences (5′-3′)
*O. tsutsugamushi*	Ot56 kD-F	CGCCAGTRATMATTCCTCCRA
	Ot56 kD-R	TTTYWGCTAGTGCRATAGAATTRG
	Taqman-Ot	FAM-TAAGGACCACACTCTAATC-MGB
*Leptospira*	Lepto F	CCCGCGTCCGATTAG
	Lepto R	TCCATTGTGGCCGR(A/G)ACAC
	Lepto P	FAM-CTCACCAAGGCGACGATCGGTAGC-BHQ
Hantavirus (HTNV)	F(771–793)	GCTTCTTCCAGATACAGCAGCAG
	R(862–884)	GCCTTTGACTCCTTTGTCTCCAT
	P(811–839)	CCTGCAACAAACAGGGAYTACTTACGGCA
Hantavirus (SEOV)	F(217–237)	GATGAACTGAAGCGCCAACTT
	R(272–291)	CCCTGTAGGATCCCGGTCTT
	P(239–263)	CCGACAGGATTGCAGCAGGGAAGAA
SFTSV (S gene)	S-F-3	GGGTCCCTGAAGGAGTTGTAAA
	S-R-3	TGCCTTCACCAAGACTATCAATGT
	S-Probe-3	TexasRed-TTCTGTCTTGCTGGCTCCGCGC-BHQ-2
SFTSV (L gene)	L-F-3	AGTCTAGGTCATCTGATCCGTTYAG
	L-R-3	TGTAAGTTCGCCCTTTGTCCAT
	L-Probe-3	HEX-CAATGACAGACGCCTTCCATGGTAATAGGG-BHQ1
SFTSV (M gene)	M-F-3	AAGAAGTGGCTGTTCATCATTATTG
	M-R-3	GCCTTAAGGACATTGGTGAGTA
	M-Probe-3	FAM-TCATCCTCCTTGGATATGCAGGCCTCA-BHQ-2

The *Y. pestis* were examined in the blood serum of the rodents samples. The V-plate method of Diagnostic Kit for *Y. pestis* F1 Antibody (Indirect Hemagglutination Assay) (Lanzhou institute biological products Co. LTD, China) was used for the detection. The blood serum was inactivated at the condition of 56°C for 30 min before the examination. First, added diluent at 25 μL/ well of the plate. Added the tested liquid 25 μL into the first well, mixed intensively and took 25 μL into the second well, mixed and diluted sequentially until the last well. F1 blood cells 25 μL were added into each well, mixed and placed at room temperature for 2 h to observe the results. 1:20 dilution of tested serum 25 μL plus negative blood cells 25 μL was as negative control. Diluted positive reference serum plus F1 blood cells 25 μL was as positive control. Diluent 25 μL plus F1 blood cells 25 μL was as blank control.

The suspected positive samples should undergo a retest test. Mix 1 portion of the tested serum with 4 portions of aldehyded blood cells, placed at room temperature for 15 min, centrifuged at 1500 rpm for 5 min, and then the supernatant (1:5) was taken for use. Two replicate tests were performed on the V-type hemagglutination plate, the first was listed as inhibition column and the second was listed as agglutination column. Added 25 μL inhibitor to each well of the inhibition column and 25 μL diluent to each well of the agglutination column. Then 25 μL of tested serum was added to the first well of each column, respectively. The two columns were diluted to the last well by multiple ratios and placed at room temperature for 15 min. F1 blood cells 25 μL were added to each well again, mixed and placed at room temperature for 3–4 h to observe the results. The positive reaction limit was based on the reaction intensity (++), and the highest dilution at which a positive reaction occurs was used as the positive titer of the serum. A positive result could be determined when the serum titer was above 1:16.

### Statistical analysis

The density of questing ticks was estimated as the number of ticks caught per flag in 100 m vegetation (ticks/flag·100 m). The density of parasitic ticks was estimated as the number of ticks caught per host animal (ticks per host animal). The rodent density was estimated as the number of rodents caught per hundred trap-nights (per 100 trap-nights). The ecological and etiological monitoring results in Zhejiang Province were described mainly by the descriptive statistics. All the descriptive statistics and plots were performed using the R version 4.0.2 (The R Foundation for Statistical Computing) with the ggmap packages.

## Results

### The density of ticks

**Figure 1:**
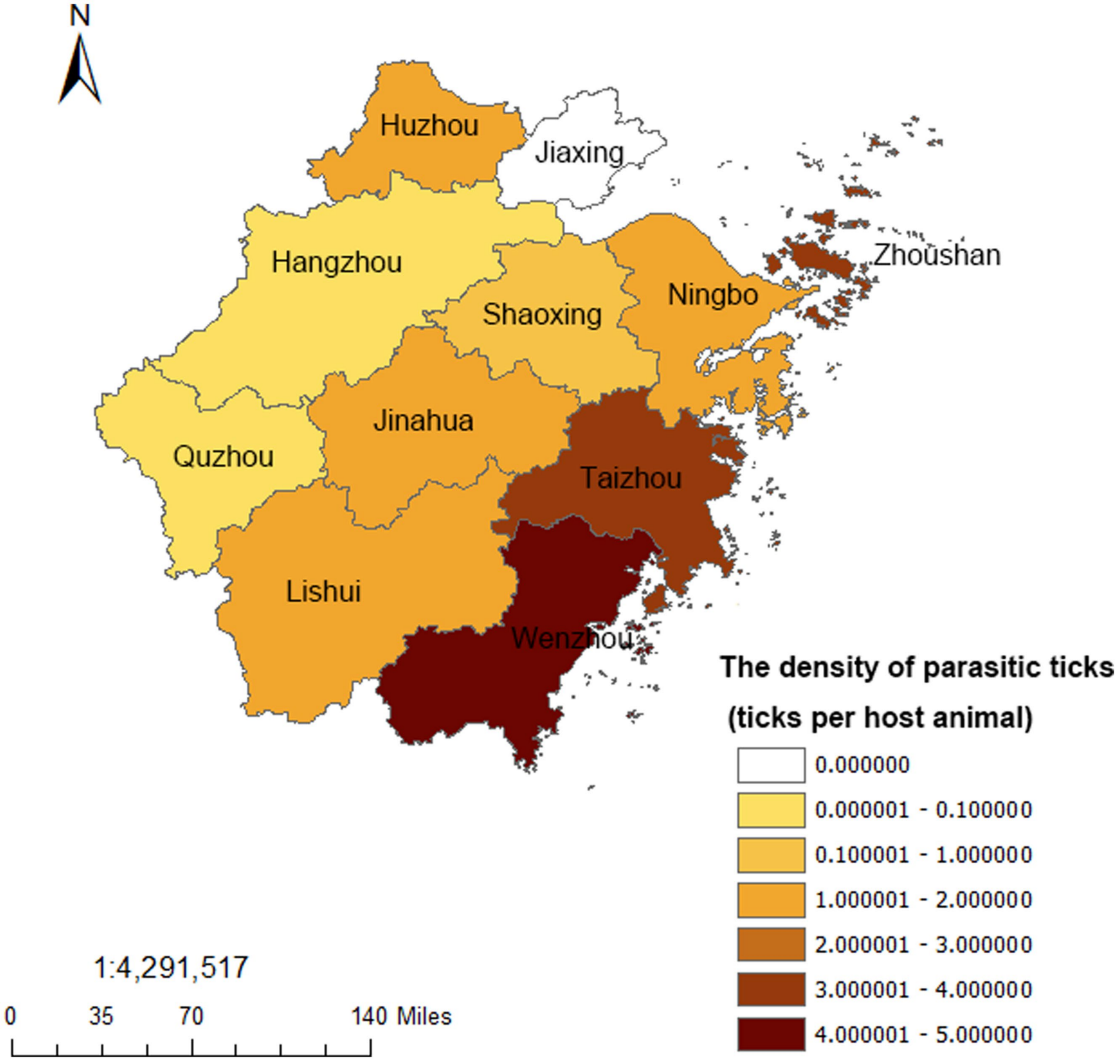
The regional distribution of the parasitic ticks from 2017 to 2022 in Zhejiang Province.

Among 12,555 animals examined in Zhejiang Province from 2017 to 2022, 16,230 parasitic ticks were found in 1848 positive animals, with the total tick density was 1.29 ticks per host animal. *H. longicornis* (74.94%) was the most abundant parasitic species in Zhejiang Province, followed by *Rhipicephalus sanguineus* (*R. sanguineus*) (10.19%) and *Rhipicephalus microplus* (*R. microplus*) (4.61%). Besides, *Rhipicephalus haemaphysaloides* (*R. haemaphysaloides*) (2.92%), *Ixodes sinensis* (*I. sinensis*) (1.73%), *Ixodes granulatus* (*I. granulatus*) (0.29%), and *Amblyomma testudinarium* (*A. testudinarium*) (0.02%) were also found. Sheep had the highest tick infection rate with a tick density of 2.78 ticks per host animal, followed by cattle (1.99 ticks per host animal). The tick infection rate of rural dogs (0.56 ticks per host animal) was slightly higher than that of urban dogs (0.21 ticks per host animal). Other animals such as rodents, chickens and ducks, etc. were also monitored in small numbers, which had the lowest parasitic tick density of 0.07 ticks per host animal (Table 2). As for the regions, the highest tick density was 4.27 ticks per host animal in Wenzhou, followed by 3.93 ticks per host animal in Taizhou and 3.39 ticks per host animal in Zhoushan. No parasitic ticks were found in Jiaxing from 2017 to 2022 (Figure 1).

**Table 2:** The monitoring results of the parasitic ticks in different host animals from 2017 to 2022 in Zhejiang Province.

Host species	No. of hosts	Positive hosts	No. of ticks	*H. longicornis*	*I. sinensis*	*A. testudinarium*	*R. haemaphysaloides*	*R. sanguineus*	*I. granulatus*	*R. microplus*	Other species	Tick density (ticks per host animal)
Cattle	1,102	245	2,194	1,137	56	0	20	99	10	602	270	1.99
Sheep	4,146	1,141	11,539	10,030	194	1	388	257	37	104	528	2.78
Rural dogs	3,006	314	1,688	852	23	2	58	677	0	28	48	0.56
Urban dogs	3,537	135	757	104	7	0	0	621	0	14	11	0.21
Other animals*	774	13	52	39	0	0	8	0	0	1	4	0.07
Total	12,555	1,848	16,230	12,162	280	3	474	1,654	47	749	861	1.29
%	—	—	—	74.94	1.73	0.02	2.92	10.19	0.29	4.61	5.30	—

*Other animals including rodents, chickens, and ducks, etc.

**Figure 2:**
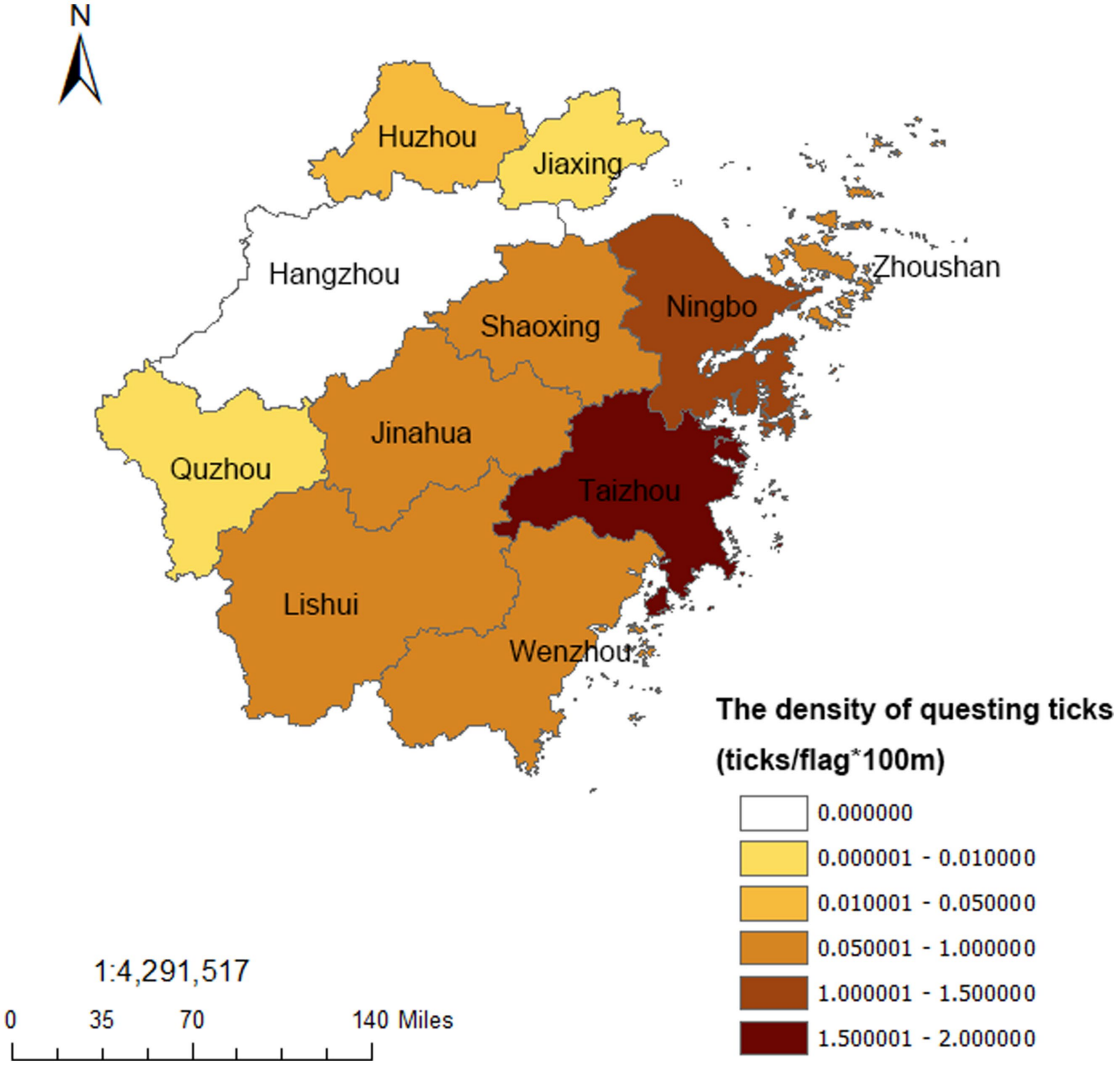
The regional distribution of the questing ticks from 2017 to 2022 in Zhejiang Province.

**Figure 3:**
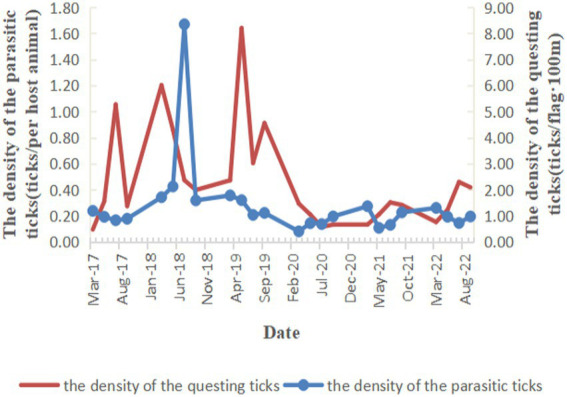
The seasonal trend of ticks density from 2017 to 2022 in Zhejiang Province.

A total of 5,201 questing ticks were captured with the tick density of 0.46 ticks/flag·100 m from 2017 to 2022 in Zhejiang Province. *H. longicornis* (87.35%) was the major species, followed by *I. sinensis* (2.19%) and *R. haemaphysaloides* (1.87%). The tick density of rural habitat was 0.82 ticks/flag·100 m, and the scenic habitat was 0.09 ticks/flag·100 m (Table 3). As for regions, the highest questing tick density was 1.72 ticks/flag·100 m in Taizhou, followed by Ningbo (1.29 ticks/flag·100 m), while the questing ticks density were lower in Jiaxing and Quzhou, and no questing ticks were found in Hangzhou (Figure 2). No obvious seasonal trend were found in both parasitic ticks and questing ticks from 2017 to 2022 in Zhejiang Province (Figure 3).

**Table 3:** The monitoring results of the questing ticks in different habitat from 2017 to 2022 in Zhejiang Province.

Habitats	Vegetation distance (m)	Total Time (min)	No. of Ticks	*H. longicornis*	*I. sinensis*	*A. testudinarium*	*R. haemaphysaloides*	*R. sanguineus*	*I. granulatus*	*R. microplus*	Others	Tick density ticks/flag·100 m
Rural habitat	569,033	34,281	4,673	4,085	97	6	80	10	0	1	394	0.82
Scenic Habitat	571,877	33,649	528	458	17	1	17	2	1	0	32	0.09
Total	1,140,910	67,930	5,201	4,543	114	7	97	12	1	1	426	0.46
%	—	—	—	87.35	2.19	0.13	1.87	0.23	0.02	0.02	8.19	—

### The density of rodents

**Figure 4:**
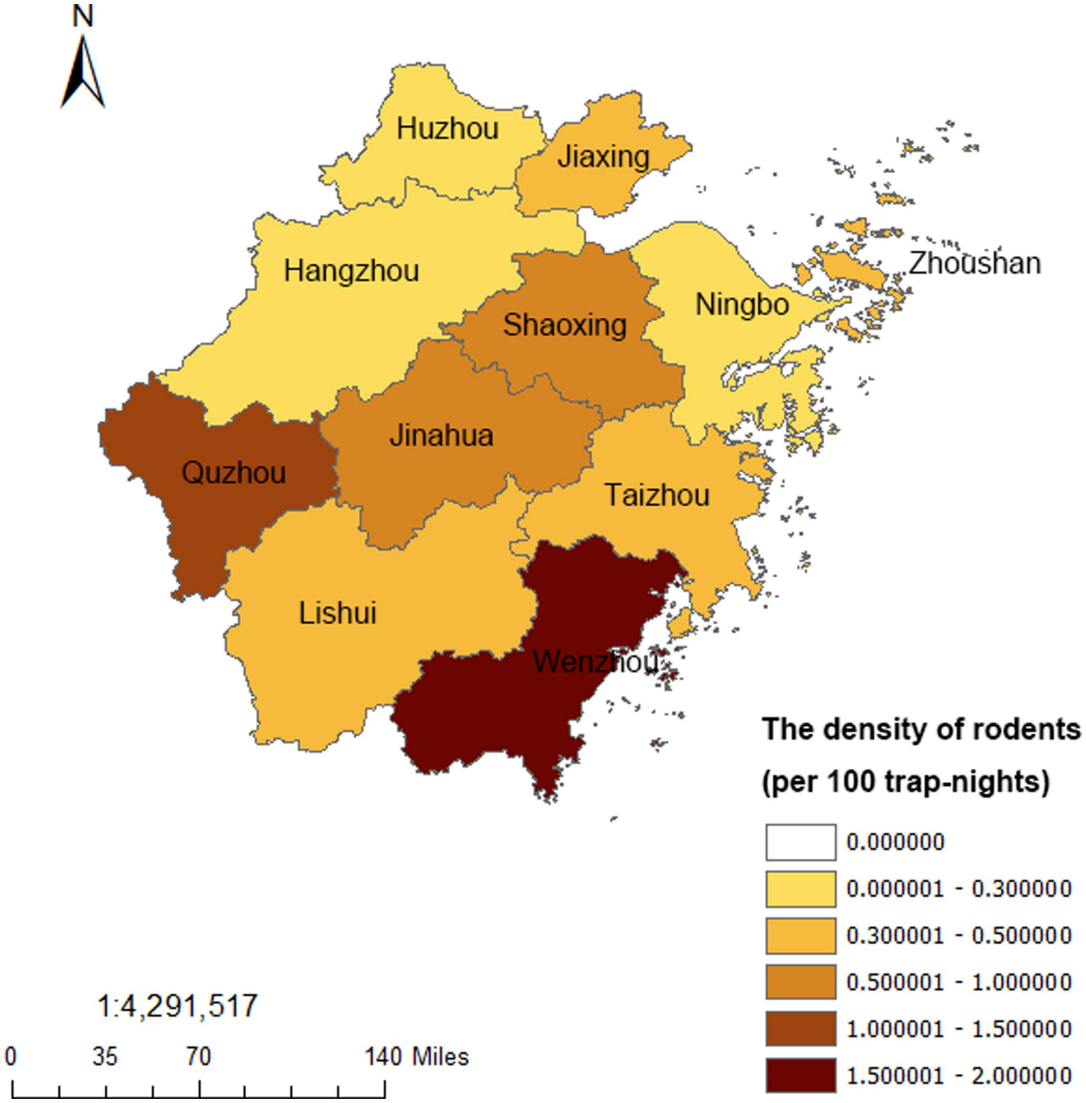
The regional distribution of the rodents from 2017 to 2022 in Zhejiang Province.

**Figure 5:**
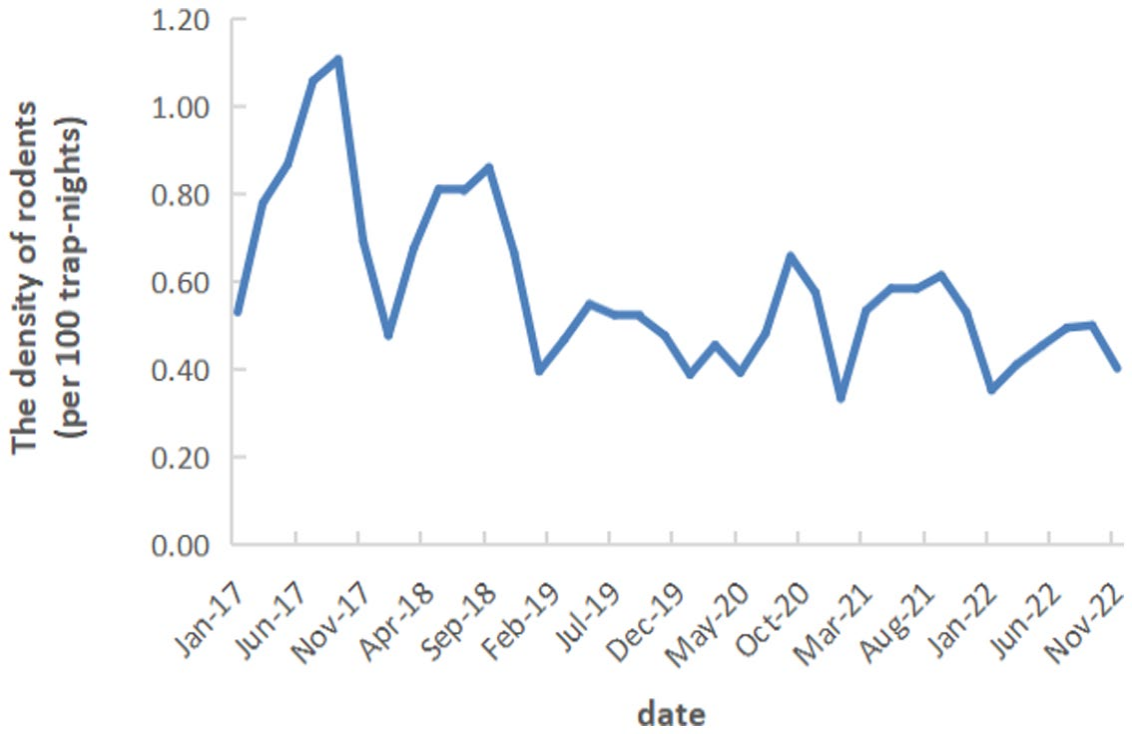
The seasonal trend in rodents density from 2017 to 2022 in Zhejiang Province.

A total of 2,187,739 mousetrap were distributed and 12,705 mice were trapped, with a density of 0.58 per 100 trap-nights from 2017 to 2022 in Zhejiang Province. The indoor rodents density was 0.72 per 100 trap-nights and the outdoor density was 0.44 per 100 trap-nights. *Rattus norvegicus* (*R. norvegicus*) was the major species (39.70%), followed by *Mus musculus* (*M. musculus*) (19.29%), *Suncus murinus* (*S. murinus*) (18.53%), *Rattus flavipectus* (*R. flavipectus*) (13.43%), and *Apodemus agrarius* (*A. agrarius*) (5.22%). Besides, *Niviventer confucianus* (*N. confucianus*), *Niviventer fulvescens* (*N. fulvescens*), *Eothenomys melanogaster* (*E. melanogaster*), *Microtus fortis* (*M. fortis*), *Micromys minutus* (*M. minutus*) etc. were also a few monitored. In different habitats, the rural residential areas had the highest density of 0.69 per 100 trap-nights, followed by the key industries (0.58 per 100 trap-nights) and urban residential areas (0.48 per 100 trap-nights) (Table 4). Among different cities, Wenzhou had the highest rodents density (1.70 per 100 trap-nights), followed by Quzhou (1.18 per 100 trap-nights), other cities all had the rodents density less than 1 per 100 trap-nights (Figure 4). In terms of the seasonal distribution, the rodents density in Zhejiang Province showed an obvious trend of seasonal fluctuation, and the peak was basically maintained in May, July, and September (Figure 5).

**Table 4:** The monitoring results of rodents in different habitat from 2017 to 2022 in Zhejiang Province.

Habitat	Mousetrap number			Rodent number			Density (per 100 trap-nights)			Rodents species													
	Indoors	Outdoors	Total	Indoors	Outdoors	Total	Indoors	Outdoors	Total	*R. norvegicus*	*M. musculus*	*S. murinus*	*R. flavipectus*	*A. agrarius*	*R. losea*	*R. nitidus*	*M. fortis*	*M. minutus*	*N. confucianus*	*E. melanogaster*	*N. fulvescens*	*R. edwardsi*	Others
Urban residential areas	309,706	427,956	737,662	2084	1,480	3,564	0.67	0.35	0.48	1,522	597	767	475	87	48	0	0	0	6	7	15	0	40
Rural residential areas	291,232	424,364	715,596	2,356	2,549	4,905	0.81	0.60	0.69	1,572	957	1,029	618	523	53	1	5	1	68	15	4	7	52
Key industries	487,182	244,004	731,186	3,397	821	4,218	0.70	0.34	0.58	1948	891	555	612	49	61	0	1	0	15	1	0	0	85
Others	1716	1,579	3,295	10	8	18	0.58	0.51	0.55	2	6	3	1	4	2	0	0	0	0	0	0	0	0
Total	1,089,836	1,097,903	2,187,739	7,847	4,858	12,705	0.72	0.44	0.58	5,044	2,451	2,354	1706	663	164	1	6	1	89	23	19	7	177
%	49.82	50.18	—	61.76	38.24	—	—	—	—	39.7	19.29	18.53	13.43	5.22	1.29	0.01	0.05	0.01	0.7	0.18	0.15	0.06	1.4

### The pathogen results of ticks

A total of 2,346 ticks were subjected for SFTSV screening, including *H. longicornis* (33.51%), *R. sanguineus* (42.20%), *R. haemaphysaloides* (13.68%), *R. microplus* (4.94%), *I. sinensis* (2.47%), *I. granulatus* (0.98%), *A. testudinarium* (0.68%), and *Dermacentor* (2.64%). 849 ticks (37.21%) were male, 873 ticks (36.19%) were female, and the rest 624 ticks (26.60%) were larval ticks. 1,312 ticks (55.92%) were collected from animals, including sheep (774 ticks), dogs (369 ticks), cattle (160 ticks) and other animals (9 ticks), and other 1,034 ticks (44.08%) were collected from vegetation.

All the ticks were grouped to 196 tubes according to the species, collection site, habitat and animals, and then tested for SFTSV. Two groups nymphal ticks of *H. longicornis* were tested to be positive, which were collected from the tea garden where the SFTS patient worked in Xinchang County of Shaoxing in 2023.

### The pathogen results of rodents

A total of 1,604 rodents were screened for SFTSV, hantavirus and *Leptospira*, 1,332 rodents were screened for *O. tsutsugamushi*, and 272 rodents were screened for *Y. pestis* in 2022 in Zhejiang Province. Among the rodents screened, 48.75% were female, 48.88% were male, and the last 2.37% were unidentified. In all the five pathogen screened, *Leptospira* had a positive rate of 12.28% in Zhejiang Province. The highest positive rate was found in Zhoushan (29.00%) and Huzhou (22.22%). The total hantavirus positive rate was 1.00%, and the highest positive rate was found in Shaoxing (9.09%). The *O. tsutsugamushi* was found positive in one rodent of Lishui (0.33%) and one rodent of Taizhou (0.33%) respectively (Table 5). No positive results were found with SFTSV and *Y. pestis* in rodents sample.

**Table 5:** The pathogen results of rodents in Zhejiang Province in 2022.

Region	Hantavirus			SFTSV			*Leptospira*			*O. tsutsugamushi*			*Y. pestis*		
	No. of rodents	No. of positive	Positive rate (%)	No. of rodents	No. of positive	Positive rate (%)	No. of rodents	No. of positive	Positive rate (%)	No. of rodents	No. of positive	Positive rate (%)	No. of rodents	No. of positive	Positive rate (%)
Hangzhou	200	0	0.00	200	0	0.00	200	0	0.00	200	0	0.00	0	0	—
Ningbo	207	0	0.00	207	0	0.00	207	14	6.76	207	0	0.00	0	0	—
Jinhua	272	1	0.37	272	0	0.00	272	6	2.21	0	0	—	272	0	0.00
Zhoushan	200	6	3.00	200	0	0.00	200	58	29.00	200	0	0.00	0	0	—
Lishui	303	0	0.00	303	0	0.00	303	46	15.18	303	1	0.33	0	0	—
Huzhou	63	3	4.76	63	0	0.00	63	14	22.22	63	0	0.00	0	0	—
Shaoxing	55	5	9.09	55	0	0.00	55	7	12.73	55	0	0.00	0	0	—
Taizhou	304	1	0.33	304	0	0.00	304	52	17.11	304	1	0.33	0	0	—
Total	1,604	16	1.00	1,604	0	0.00	1,604	197	12.28	1,332	2	0.15	272	0	0.00

*Leptospira* was tested to be positive in almost all the main rodent species except for *N. fulvescens* in Zhejiang Province, and *R. losea* (22.22%), *R. norvegicus* (16.17%), *A. agrarius* (16.15%), *Berylmys bowersi* (*B. bowersi*) (14.29%) and *S. murinus* (12.94%) all had a positive rate over 10% (Table 6). The habitats of the positive rodents were distribution in the farmland (38.07%), the village (32.99%), the mountainous region and shrubbery (10.66%), the key industries (10.15%), and the urban residential areas (8.12%) (Table 7). Hantavirus was tested to be positive in *R. norvegicus* (2.26%), *M. musculus* (1.54%), *S. murinus* (1.18%), *R. flavipectus* (0.53%) and *A. agrarius* (0.26%), and the habitat of positive rodents were the key industries (43.75%), the village (37.5%), the urban residential areas (6.25%), the farmland (6.25%), and the mountainous region and shrubbery (6.25%) (Table 7). *O. tsutsugamushi* was only screened positive in two *A. agrarius* (0.69%) which were captured from the farmland.

**Table 6:** The pathogen results of different rodent species in Zhejiang Province in 2022.

Rodent species	Hantavirus			SFTSV			*Leptospira*			*O. tsutsugamushi*			*Y. pestis*		
	No. of rodents	No. of positive	Positive rate (%)	No. of rodents	No. of positive	Positive rate (%)	No. of rodents	No. of positive	Positive rate (%)	No. of rodents	No. of positive	Positive rate (%)	No. of rodents	No. of positive	Positive rate (%)
*R. norvegicus*	532	12	2.26	532	0	0	532	86	16.17	505	0	0	27	0	0
*A. agrarius*	390	1	0.26	390	0	0	390	63	16.15	289	2	0.69	101	0	0
*R. flavipectus*	190	1	0.53	190	0	0	190	5	2.63	174	0	0	16	0	0
*E. melanogaster*	108	0	0	108	0	0	108	3	2.78	1	0	0	107	0	0
*S. murinus*	85	1	1.18	85	0	0	85	11	12.94	85	0	0	0	0	—
*N. confucianus*	79	0	0	79	0	0	79	7	8.86	68	0	0	11	0	0
*M. musculus*	65	1	1.54	65	0	0	65	3	4.62	64	0	0	1	0	0
*R. losea*	63	0	0	63	0	0	63	14	22.22	63	0	0	0	0	—
*R. edwardsi*	36	0	0	36	0	0	36	1	2.78	34	0	0	2	0	0
*M. fortis*	30	0	0	30	0	0	30	3	10	30	0	0	0	0	—
*N. fulvescens*	13	0	0	13	0	0	13	0	0	6	0	0	7	0	0
*B. bowersi*	7	0	0	7	0	0	7	1	14.29	7	0	0	0	0	—
Others	6	0	0	6	0	0	6	0	0	6	0	0	0	0	—
Total	1,604	16	1	1,604	0	0	1,604	197	12.28	1,332	2	0.15	272	0	0

**Table 7:** The habitats of the positive rodents in Zhejiang Province in 2022.

Pathogen	Rural residential areas						Key industries		Urban residential areas		Total
	Farmland		Village		Mountainous region and shrubbery						
	No. of rodents	%	No. of rodents	%	No. of rodents	%	No. of rodents	%	No. of rodents	%	No. of rodents
*Leptospira*	75	38.07	65	32.99	21	10.66	20	10.15	16	8.12	197
hantavirus	1	6.25	6	37.50	1	6.25	7	43.75	1	6.25	16
*O. tsutsugamushi*	2	100.00		0.00		0.00		0.00		0.00	2
Total	78	36.28	71	33.02	22	10.23	27	12.56	17	7.91	215

## Discussion

In our study, with the wide coverage and representativeness to Zhejiang Province, the ecological and etiological monitoring of ticks and rodents were conducted. Findings showed that *H. longicornis* and *R. norvegicus* was the major tick species and rodents species in Zhejiang Province, respectively. SFTSV was found to be positive in ticks samples, and *Leptospira*, hantavirus and *O. tsutsugamushi* were found to be positive in rodents samples.

Ticks, especially the *H. longicornis*, which had a higher density and wider distribution, were suggested to be the primary vector of SFTSV in China in recent years (1–4). Research found that the tick density was one of the important factors affecting the occurrence of SFTS, and a high tick density reminded a higher exposure risk to SFTS (24, 25). Zhejiang Province has always been the natural epidemic focus of SFTS (26). Wu et al. found that 37 counties, including 118 towns, were affected by SFTS during 2011–2018 in Zhejiang Province and the numbers of affected counties increased year by year (27). Li et al. also detected three significant clusters, which accounted for 53.61% of total SFTS cases in Zhejiang Province (28). In our study, areas with higher tick density in the east area of Zhejiang Province such as Taizhou, Zhoushan, Ningbo were coincided with those which had a higher SFTS incidence in these researches (27, 28), suggesting the tick density surveillance could play an important role in the SFTS surveillance and early warning. Relevant researches found that SFTS was mostly endemic in rural areas, and the cases were more likely to be farmers (29, 30). Tick bites, as well as raising domestic animals, grazing, farming, presence of rats, or contact with wild animals were risk factors for SFTSV infection (31, 32). In this study, we found a higher tick density in rural habitat than scenic habitat, the highest tick infection rate was in sheep, and the rural dogs had a higher tick infection rate than urban dogs. These results all indicated that the farmers had a greater exposure to ticks for the agricultural activity or raising animals, which might be one reason for the high infection rate of SFTS. Besides, the positive results of two groups of *H. longicornis* collected from the tea garden where the SFTS patient worked in our research also added some supports.

In this study, the rodents had a density of 0.58 per 100 trap-nights in Zhejiang Province from 2017 to 2022, which was relatively lower than other provinces which was in the north of China (33). We found that *R. norvegicus* was the major species, followed by *M. musculus*, *S. murinus*, and *R. flavipectus*, and the indoor rodents density was higher than that of outdoor density, indicated a high invasion rate of rodents to human habitation, which provided favorable conditions for the spread of rodents-borne diseases, such as HFRS, leptospirosis, and scrub typhus, etc. (11, 15, 20). Based on the results of the national surveillance report on rodent-borne pathogens of disease vectors in 2021 of China, most pathogens were detected in the farmland (7 species pathogens) and the village (7 species pathogens), and 1–4 species pathogens were detected in other habitats such as urban residential areas and the key industries (34). With the highest rodent density found in our results and the relative pathogen detection, the rodent-borne diseases in rural areas required further attention. A prior study found that the rodent density showed geographical autocorrelation, and counties with a higher rodent density were mainly distributed in southern area of Zhejiang Province (35), while in our study, Wenzhou and Quzhou had the highest rodents density above 1 per 100 trap-nights, which was consistent with these previous findings to some extent. Previous study had shown that the density of rodents increased during the spring breeding season, and experience two unconspicuous density peaks in spring and autumn in Zhejiang Province (35). While in our study, the seasonal fluctuation of rodents was obvious increased from the spring, but the peak was maintained in May, July, and September, and the two density peaks was not observed clearly.

Among the five pathogens screened in rodents, we found that *Leptospira* had the highest detection rate of 12.28% in Zhejiang Province. Except for Hangzhou City, the other 7 prefecture-level cities all had positive results, and two cities were even found had the positive rate over 20%, indicating that *Leptospira* was widespread in the form of high infection rate in Zhejiang Province. A literature review found that overall, 30.3% of *R. norvegicus*, 19.3% of *R. argentiventer*, 17.8% of *R. rattus*, 13.1% of *R. losea*, 10.9% of *R. exulans*, and 3.4% of *R. tanezumi* were reported to be positive for *Leptospira* (11). In our study, *Leptospira* was tested to be positive in almost all the common rodent species in Zhejiang Province, and the major species rodents including *R. losea*, *R. norvegicus*, *A. agrarius*, *B. bowersi*, and *S. murinus* all had the positive rate over 10%, suggesting a very serious infection rate in the rodents population. The positive rodents were widely distributed in almost all the habitats including farmland, the village, the mountainous region, the shrubbery, the key industries, and the urban residential areas, which were closely connected with human population to realize the spread of diseases from rodents to human easily.

In our study, the total hantavirus positive rate in rodents was 1.00% in Zhejiang Province, and five of the eight cities were tested to be positive. The major rodents detected positive including *R. norvegicus*, *M. musculus*, *S. murinus*, *R. flavipectus* and *A. agrarius*, and the relevant research found that *A. agrarius* and *R. norvegicus* were their major hosts (36). The positive rodents were widely distributed in almost all the monitoring habitats, which also hinted a risk of the disease transmission. The *O. tsutsugamushi* was found positive in only two *A. agrarius* captured from the farmland, and the two rodents were collected from Lishui and Taizhou City, respectively. A previous study found that the positive rate of *O. tsutsugamushi* was 0.35% in Zhejiang Province in 2020 (23), which was generally consistent with our results. Zhejiang Province was the plague epidemic area in history, and one research found that 3 *A. agrarius* samples were tested positive of plague F1 antibody test in Longquan and Yiwu City in 2005 (10). But no positive result was found with *Y. pestis* in 2022 in our analysis. Considering the severity of the impact on human health and the prolonged epidemic character, the surveillance of plague requires continuous attention in further study.

SFTSV was supposed to circulate in an enzootic tick-vertebrate-tick cycle, yet the vertebrate hosts in nature had not been confirmed. Although rodents were suspected to be the reservoir hosts of Bunyaviruses, and a previous study also found there might be pronounced discrepancy on SFTSV in rodents, there was not enough evidence to confirm the rodents were reservoir hosts (25). A study showed the pooled seroprevalence of anti-SFTSV antibodies was 3.20% in rodents (37), and another study found the positive result of SFTSV RNA was 0.7% in rodents (38). In our study, no positive results were found in the 1,604 rodents in 8 prefecture-level city with SFTSV. Whlie Ni et al. successfully detected SFTSV RNA in 2 of 8 *A. agrarius* in Ningbo City of Zhejiang Province, and found the SFTSV segments isolated from the rodents shared great sequence homologies to those isolated from the patients living in nearby villages (39). So the SFTSV might be present in rodents of Zhejiang Province, and the rodents might be one of the natural hosts of SFTSV. The negative results of our research might be due to the low carrier rate of the SFTSV in the rodents population in these area, and further researches should be conducted.

## Conclusion

In conclusion, the ticks and rodents were widely distributed in Zhejiang Province, and the density of both vectors were high in certain areas. The SFTSV were tested positive in the ticks of *H. longicornis*, suggesting a risk of the spread of SFTS. The *Leptospira* had a very high positive rate in the rodents samples in Zhejiang Province. Besides, the hantavirus and the *O. tsutsugamushi* were also examined positive in the rodents samples, indicating that this should be a public health problem deserving more attention in the rodents-borne disease. Furthermore, the prevention and control of the relevant disease of both ticks and rodents need more further study.

## Data availability statement

The original contributions presented in the study are included in the article/supplementary material, further inquiries can be directed to the corresponding authors.

## Ethics statement

Ethical approval was not required for the study involving animals in accordance with the local legislation and institutional requirements because the ethics committee approved the procedure for verbal consent because Zhejiang CDC has the authority of the Zhejiang provincial government to collect the related information, which is part of the disease surveillance work. This surveillance mainly involved the density and etiological detection of wild mice and ticks, and documentation of consent was not required.

## Author contributions

JW: Writing – original draft. ML: Writing – original draft. TL: Writing – review & editing. YL: Writing – review & editing. GJ: Writing – review & editing. YW: Writing – review & editing. QL: Writing – review & editing. ZG: Writing – review & editing. JS: Writing – review & editing.
